# Supplementary figures and images for: Meloxicam ameliorates the cartilage and subchondral bone deterioration in monoiodoacetate-induced rat osteoarthritis
Source: PeerJ. 2017 Apr 12;5:e3185. doi: 10.7717/peerj.3185 (PMC5391791; doi:10.7717/peerj.3185)

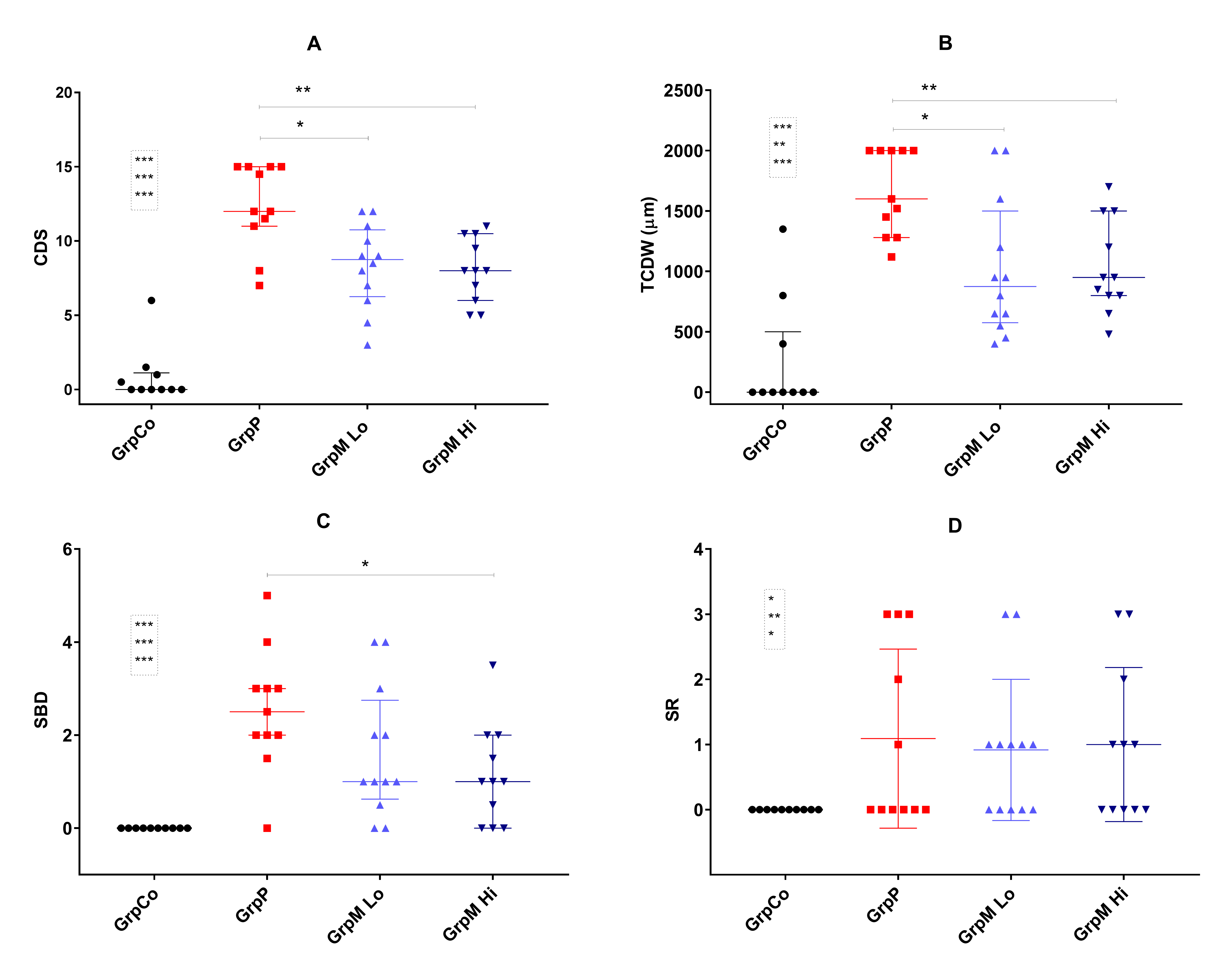

Supplement: Supplemental Information 1 — (A) CDS, cartilage degeneration score; (B) TCDW, total cartilage degeneration width; (C) SBD, calcified cartilage and subchondral bone damage score; (D) SR, synovial reaction. Values shown as median and interquartile range. Significant differences marked with * for p < 0.05, ** p < 0.01. Comparisons for GrpCo shown in boxes, in order for GrpP, GrpM Lo, GrpM Hi. [file peerj-05-3185-s001.png]
