# Supplementary material for: Meloxicam ameliorates the cartilage and subchondral bone deterioration in monoiodoacetate-induced rat osteoarthritis
Source: PeerJ. 2017 Apr 12;5:e3185. doi: 10.7717/peerj.3185 (PMC5391791; doi:10.7717/peerj.3185)
Supplement: Table S1 — Values expressed as mean with median, minimum– maximum values in brackets. Holm-Bonferroni adjusted * p < 0.05, **, ††p < 0.01 for paired comparisons between groups II and III (marked with *), and groups II and IV (marked with †), respectively. TCDW given in μm. Significances for comparisons of Group I are not shown. Scores are those of the OARSI histopathology initiative: CDS, cartilage degeneration score; TCDW, total cartilage degeneration width; SBD, calcified cartilage and subchondral bone damage score; SR, synovial reaction. [file peerj-05-3185-s002.docx]

| Score | GrpCo  (n=10) | GrpP  (n=11) | GrpM Lo  (n=12) | GrpM Hi  (n=11) | |
| --- | --- | --- | --- | --- | --- |
| CDS | 0.9 (0, 0-6) | 12.36 (12, 7-15)**^, ††^ | 8.33 (8.75, 3-12)** | 8.05 (8, 5-11)**^, ††^ | |
| TCDW | 255 (0, 0-1350) | 1659 (1600, 1120- 2000)**^, ††^ | 1016 (875, 400- 2000)** | 1034 (950, 480-1700)**^, ††^ | |
| SBD | 0 (0, 0-0) | 2.54 (2.5, 0-5)* | 1.62 (1, 0-4) | 1.13 (1, 0-3.5)* | |
| SR | 0 (0, 0-0) | 1.09 (0, 0-3) | 0.91 (1, 0-3) | 1.00 (1, 0-3) | |
|  | | | | |  |
